# Lung Metastases in Patients with Stage IV Pancreatic Cancer: Prevalence, Risk Factors, and Survival Impact

**DOI:** 10.3390/jcm8091402

**Published:** 2019-09-06

**Authors:** Keng-Hao Liu, Chia-Yen Hung, Shu-Wen Hsueh, Pei-Hung Chang, Yen-Yang Chen, Chang-Hsien Lu, Ping-Tsung Chen, Kun-Yun Yeh, Pei-Wei Huang, Ngan-Ming Tsang, Yu-Shin Hung, Wen-Chi Chou

**Affiliations:** 1 Department of Surgery, Chang Gung Memorial Hospital at Linkou, Linkou 333, Taiwan; 2 Department of Hematology-Oncology, Chang Gung Memorial Hospital at Linkou, Linkou 333, Taiwan; 3 Division of Hematology-Oncology, Department of Internal Medicine, Mackay Memorial Hospital, Taipei 104, Taiwan; 4 Division of Hematology-Oncology, Department of Internal Medicine, Chang Gung Memorial Hospital at Keelung, Keelung 204, Taiwan; 5 Division of Hematology-Oncology, Department of Internal Medicine, Chang Gung Memorial Hospital at Kaohsiung, Kaohsiung 833, Taiwan; 6 Division of Hematology-Oncology, Department of Internal Medicine, Chang Gung Memorial Hospital at Chiayi, Chiayi 612, Taiwan; 7 Department of Radiation Oncology, Chang Gung Memorial Hospital at Linkou, Linkou, 333, Taiwan

**Keywords:** pancreatic cancer, lung metastases, incidence, risk factor, outcome, palliative chemotherapy

## Abstract

The aim of this study was to evaluate the prevalence, the clinicopathological variables associated with probability of lung metastases, and the impact of lung metastases on survival outcome in patients with stage IV pancreatic cancer (PC) treated with palliative chemotherapy. A total of 654 patients with stage IV PC who underwent palliative chemotherapy from 2010–2016 were retrospectively enrolled in this study. Possible clinical variables associated with lung metastases and survival outcome were examined by univariate and multivariate analysis. Lung metastases were detected in 15.0% (3.4% with isolated lung metastases and 11.6% with synchronic metastases to lung and other organs). Female gender, poorly differentiated tumor grade, and large primary tumor size were independent risk factor in multivariate analysis. The median overall survival (OS) time was 6.5 months in the entire cohort, while the median OS was 11.8, 6.9, 7.7, 10.1, and 5.0 months for patients with isolated lung, isolated liver, isolated peritoneum, isolated distant lymph nodes, and multiple sites metastases, respectively. Isolated lung metastases were a better prognosticator for OS in univariate and multivariate analysis. This study utilized real-world clinical practice data to assess the prevalence, risk factors, and survival impact of lung metastases in patients with stage IV pancreatic cancer.

## 1. Introduction

Pancreatic cancer (PC) is one of the most lethal cancers, accounting for 3.2% of newly diagnosed malignancies and 7.5% of cancer-related deaths in the United States in 2019 [1]. In Taiwan, PC accounted for 2.1% of newly diagnosed malignancies and 4.1% of the cancer-related deaths in 2016 [2]. At the time of PC diagnosis, approximately 50% of patients presented with stage IV disease [1], while 19–39% of these patients presented with lung metastasis. [3,4]. Despite the median survival time of around 6–11 months in patients with metastatic PC [3,4,5], several studies reported that isolated lung metastases are associated with better survival outcome than for patients presenting with other solitary metastatic organs [6,7,8,9,10,11,12,13]. However, these studies were limited by the small patient sample size [7,8,9,10,11,12,13], designed for patients who received specific treatment modality (mainly surgical resection) [7,8], or selected from a single institute that would not be applicable worldwide [7,8,9,10,11,12,13]. Furthermore, none of the studies have explored the association of clinicopathological variables with lung metastases in PC patients. Here we conducted a multi-institute study to evaluate the prevalence, the clinicopathological variables associated with probability of lung metastases, and the impact of lung metastases on survival outcome in patients with stage IV PC treated with palliative chemotherapy.

## 2. Patients and Methods

### 2.1. Patient Selection

We conducted a retrospective review that included a total of 746 patients with metastatic PC who underwent palliative chemotherapy from 2010–2016 at four medical centers in Taiwan. All patients were selected from the institutional cancer registry center and had either pathologically or radiographically diagnosed stage IV disease. Diagnosis of pancreatic cancer was based on the pathological report. Metastatic organs involved were found by image studies with either computerized tomography scan or magnetic resonance imaging scan, and further confirmed by a multidisciplinary tumor board. Pathological diagnosis of lung metastasis was mandatory for patients presenting with isolated lung nodules. Patients who had active concurrent malignancy (*n* = 23), had incomplete information on the medical charts, for example, missing record of comorbidity, performance status, or lost follow up (*n* = 56), or could not rule out primary lung cancer by pathologic or image study (*n* = 13), were excluded. The remaining 654 patients were enrolled for final analysis. All patients were further categorized into isolated lung, isolated liver, isolated peritoneum, isolated distant lymph nodes, and multiple metastatic sites according to involvement of metastatic organs for survival analysis. The isolated metastases were defined as presence of tumor metastasis/metastases limited to solitary organ. Distant lymph nodes were defined as those other than the regional lymph nodes according to the 7th edition of AJCC staging system for exocrine pancreatic cancer [14]. Metastatic tumors which involved more than one organ were classified as multiple sites metastases. This study was approved by the institutional review boards of all the participating institutes and was conducted in compliance with the Helsinki Declaration (1996).

### 2.2. Data Collection

Using a prospectively formulated electronic data form from our previous research [15,16,17], primary care physicians recorded the patients’ demographic and clinical data including age, sex, body mass index, Eastern Cooperative Oncology Group performance status (ECOG PS), smoking history, pre-existing comorbidities, anatomic location of the primary cancer, clinical stage as determined using the 7th edition of the American Joint Committee on Cancer (AJCC), serum carcinoembryonic antigen (CEA) and carbohydrate antigen 19-9 (CA19-9) levels, organ of metastatic site, and chemotherapy regimens at the time before initiation of antitumor therapy. The chemotherapeutic agent, dosage, and treatment schedule were determined by the primary care physician. Overall survival (OS) was calculated from the date of initiation of palliative chemotherapy to the date of death from any cause or 31 December 2017. All dates of death were obtained from either the Institutional Cancer Registry or the National Registry of Death in Taiwan.

### 2.3. Statistical Analysis

Basic demographic data were summarized as n (%) for categorical variables and median with range for continuous variables. Univariate and multivariate analysis of overall survival for all clinical factors was performed using the log-rank test. Possible clinical variables associated with lung metastases among PC patients were examined by univariate and multivariate logistic regressions. All of the variables in univariate analysis with *p* values <0.10 were further analyzed using a multivariate analysis. Survival time was analyzed using the Kaplan–Meier method. Log-rank tests were used to determine the significance of differences between the survival curves. SPSS 17.0 software (SPSS Inc., Chicago, IL, USA) was used for statistical analysis. All statistical assessments were two-sided, and a *p*-value less than 0.05 was considered statistically significant.

## 3. Results

The demographic characteristics of 654 patients are shown in Table 1. The median age was 62 (range, 25–88) years, and 393 (50.1%) patients were men. The distribution of patients with number of metastatic organs of 1, 2, 3 and ≥4 was 391 (59.8%), 191 (29.2%), 53 (8.1%), and 19 (2.9%) of the total patients, respectively. The most common metastatic organs were liver (67.0%), followed by peritoneum (36.5%), distant lymph nodes (22.9%), and lung (15%). Twenty-three (3.5%) patients had previously received pancreatectomy at diagnosis of metastatic disease.

**Figure 1:**
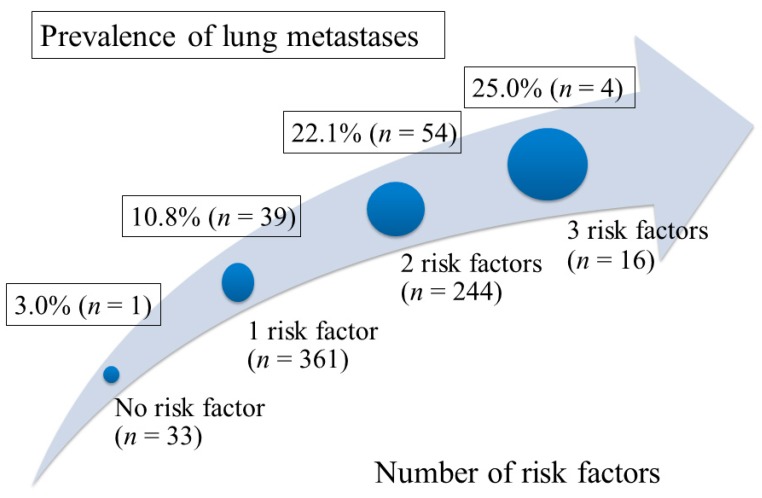
The prevalence of lung metastases according to the number of risk factors present in the patients. Risk factors for occurrence of lung metastases included female gender, poorly differentiated tumor grade, and primary tumor size ≥8 cm.

Lung metastases were detected in 98 (15.0%) of 654 patients, including 22 (3.4%) patients with isolated lung metastases and 76 (11.6%) patients with synchronic metastases to lung and other organs. In univariate analysis, the clinical factors associated with higher probability of lung metastases included female gender (19.9% vs. 11.7% in male, HR (hazard ratio) 1.88; 95% confidence interval (CI), 1.22–2.89; *p* = 0.004), poorly differentiated or undifferentiated tumor grade (16.1% vs. 4.8% in well or moderately differentiated tumor grade, HR 3.83, 95% CI, 1.18–12.5; *p* = 0.026), and primary tumor size ≥8 cm (26.7% vs. 14.1% in tumor size <8 cm, HR 2.21; 95% CI, 1.10–4.45; *p* = 0.031). Gender, tumor grade, and primary tumor size remained as independent risk factors associated with high probability of lung metastases in multivariate analysis (Table 2). These three risk factors are further analyzed by calculating the probability of lung metastases according to the number of risk factors present in PC patients as shown in Figure 1. For patients who presented with 0, 1, 2, and 3 risk factors, the lung metastasis probability rate was 3.0% (1 of 33), 10.8% (39 of 361), 22.1% (54 of 244), and 25.0% (4 of 16), respectively.

**Figure 2:**
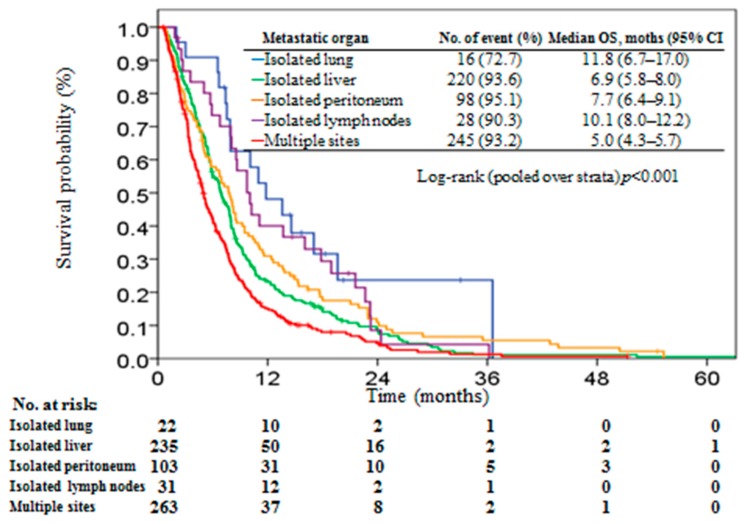
Kaplan–Meier plot of overall survival in stage IV pancreatic patients stratified by metastatic organ.

Overall, the median survival time was 6.5 months (95% CI, 5.9–7.2) and 607 (92.8%) patients had died at the end of the study. The distribution of patients with isolated lung, isolated liver, isolated peritoneum, isolated distant lymph nodes, and multiple sites were 3.4%, 36.0%, 15.7%, 4.7%, and 40.2%, respectively, of the total patients, and the median OS was 11.8 (95% CI, 6.7–17.0), 6.9 (95% CI, 5.8–8.0), 7.7 (95% CI, 6.4–9.1), 10.1 (95% CI, 8.0–12.2), and 5.0 (95% CI, 4.3–5.7) months, respectively (Figure 2). A significant survival difference was observed among patients with different metastatic organ groups (log-rank *p* < 0.001). Patients with isolated lung metastases had significant survival outcome than those with isolated liver (*p* = 0.005) and multiple site metastases (*p* < 0.001). Although patients with isolated lung metastases had longer survival time than those with isolated peritoneum and isolated distant lymph node metastases, the survival outcome did not reach significant difference (*p* = 0.07 and 0.33, respectively).

Univariate and multivariate analyses of clinical variables associated with OS are presented in Table 3. Female gender, age ≤ 65 years, better performance with ECOG score 0 or 1, isolated lung metastases, and low comorbidity with CCI 0 to 1 were significant positive prognostic factors for OS in univariate analysis. Isolated lung metastases remained as an independent good prognosticator for OS in multivariate analysis.

## 4. Discussion

The impact of lung metastases on survival outcome in PC patients has not been comprehensively investigated. While few studies have addressed favorable survival outcome from surgical resection of isolated lung metastasis [7,8], the survival impact of lung metastases in PC patients treated with palliative chemotherapy remained unclear. To the best of our knowledge, this is the largest observational cohort study to evaluate the incidence, risk factors, and survival impact of lung metastases in patients with stage IV PC. In our study, lung metastases were detected in 15.0% of 654 patients, including 3.4% of patients with isolated lung metastases and 11.6% of patients with synchronous metastases to lung and other organs. We identified that female gender, primary pancreatic tumor size ≥8 cm, and poorly differentiated/undifferentiated histological tumor grade were independent predictors for the presence of lung metastases in patients with stage IV PC.

The survival outcome from this study concurred with previous studies with a median OS within the 6–11 month range reported for stage IV pancreatic cancer patients treated with palliative chemotherapy [3,4,5]. Studies demonstrating favorable survival outcome of isolated lung metastases in patients with pancreatic cancer were all involved with surgical resection of the metastatic lung lesion [7,8]. While our study focused on PC patients with isolated lung metastases who receive antitumor treatment with palliative chemotherapy for lung metastasis, results revealed better survival outcome compared to patients with other solitary metastatic site or multiple metastatic sites.

The impact of lung metastasis on survival outcomes in patients receiving non-surgical treatment modality had not been extensively explored. Kruger et al. reported a median survival of 22.5 months for 40 patients with pancreatic cancer and isolated lung metastases receiving palliative chemotherapy with mainly gemcitabine-based regimen [9]. The authors identified that patients with less than 10 lung metastases (31.3 months) or unilateral localization of lung metastases (31.3 months) were better prognostic groups than the other patient groups (18.7–21.8 months). Decoster et al. reported a case-control study comparing survival outcome of 37 pancreatic cancer patients with isolated lung metastases to 37 patients with isolated liver metastases receiving treatment with palliative chemotherapy [10]. Patients with isolated lung metastases had significantly better median survival time compared to those with isolated liver metastases (20.8 months vs. 9.1 months, *p* < 0.001). Due to the different survival outcomes among patients with different clinical scenarios of lung metastases, Lovecek et al. divided patients with lung metastases into three subgroups for survival comparison: isolated oligometastases of lung had better outcome than isolated multiple metastases of lung, whereas worst outcome was in patients with lung metastases accompanied with other organ metastases [11], yet the in-groups difference of survival outcome had not been validated externally. Our data were consistent with previous reports, which presented that isolated lung metastases had better survival outcome than those with synchronous metastases of other organs.

Next to liver, peritoneum, and distant lymph nodes, the lung is the fourth most common metastatic organ site for PC, accounting for 15.0% of patients with stage IV pancreatic cancer in our study and 19–39% of patients with stage IV PC in the Western population [3,4]. However, the actual incidence of lung metastases is believed to be underestimated due to nonspecific symptoms in the early phase of lung metastases and lack of consensus among physicians for the comprehensive lung filed image study as tumor staging for PC. Although PC staging with computed tomography (CT) scan, including abdomen and whole lung field, is recommended by the 8th version of the AJCC cancer staging manual [18], the clinical utility of CT scan including the whole lung field is still debated [19]. Firstly, isolated lung metastasis is a relatively rare entity, accounting for 3.4% of 623 patients with pancreatic cancer in a study by Mehtsun et al. [20] and 3.4% of current study. As lung metastases were often accompanied with other organs’ metastases, discovery of lung metastases seldom changes the fact of M1 disease [21]. Second, while CT has high sensitivity to identifying indeterminate lung nodules (ILNs), the noncalcified lung nodules ≤1 cm in diameter, the low specificity of CT scan for metastatic lung nodule brings more queries than directions to approaches of the possible metastatic site [22,23]. Poruk et al. reported that 183 (49%) of 374 PC patients had ≥1 ILNs at preoperative chest CT scan [22]. Their study revealed ILN as a lung metastasis in only one of six patients with pancreatic cancer who were undergoing lung resection. Most importantly, median survival outcome was comparable among patients with and without ILN treated with pancreatectomy. Similar, Chang et al. reported that ILN was identified in 59 (18%) of 329 patients receiving preoperative chest CT scan before pancreatic cancer resection, while only 5 (1.5%) patients were pathologically diagnosed with lung metastases in subsequent follow-up [23]. Similarly, there was no survival difference between patients with and without preoperative ILNs. Our data suggested that a chest CT scan might provide more comprehensive tumor staging for a small subset of patients with PC (3.4% of patients with isolated lung metastases in our cohort). In addition, our study highlighted the importance of identifying the clinicopathological factors associated with lung metastases probability, maximizing the cost-performance value of chest CT scan in PC patients.

Identification of the clinicopathological variables associated with higher probability of lung metastases is important for accurate tumor staging and appropriate treatment strategy. This study revealed gender, histological differentiation, and primary pancreatic tumor size as independent predictors for lung metastases in PC patients. Our study showed the probability of lung metastases increased dramatically from presence of none of the risk factor (3.0%) to presence of all three risk factors (25%). Poorly differentiated histology and large tumor size indicate aggressive tumor behavior, which contributes to the higher probability of distant metastases. Interestingly, our study showed that the female gender has more occurrence of lung metastases than the male gender. Our previous study demonstrated that the female gender had better survival outcome than the male gender in patients with stage IV pancreatic cancer [24]. Similarly, Oweira et al. reported that the female gender is a better independent prognosticator based on 13,233 patients with stage IV pancreatic cancer from the SEER database [6]. The better prognosis in patients with female gender might be partially attributed to the better treatment response to chemotherapy [25] along with the higher probability of isolated lung metastases in female patients with advanced pancreatic cancer. These observations suggest intrinsic pathogenesis differences of gender in pancreatic cancer that contribute to distinct clinical presentation and survival outcome [25]. Future study is necessary to select a patient subgroup with lung metastases that might have the most favorable outcome undergoing the palliative chemotherapy.

This multi-institutional study was strengthened by a large patient sample size studied across a 7-year duration, which may be an adequate representation of real-world clinical care for pancreatic cancer. We reported the incidence, risk factors, and impact of lung metastases in patients with stage IV pancreatic cancer treated with palliative chemotherapy that might informative for physicians and patients. However, this study had some limitations. First, due to the retrospective nature of this study, selective bias exists. Second, the prevalence of patients with isolated lung metastases is low, which might diminish the statistical power in multivariate analysis. Third, the incidence of lung metastases among patients with multiple organ synchronous metastases might be overestimated in our study group because some ILNs may be misread as metastatic lesions. Fourth, the probability risk factors for lung metastases were based on retrospective evaluation of the situation at the time of the initiation of palliative chemotherapy, as this may differ by the time of diagnosis. Finally, our study only included patients who underwent palliative chemotherapy, while patients who did not receive the treatment were excluded, and as such there was selection bias.

## 5. Conclusions

This study utilized real-world clinical practice data to assess the prevalence, risk factors, and survival impact of lung metastases in patients with metastatic PC. Our findings indicate that the lung is a common metastatic site of pancreatic cancer, while patients with female gender, large primary tumor, and poorly differentiated histological tumor grade had the highest probability of lung metastases. Our study showed patients with isolated lung metastases had better survival outcomes than patients with metastasis to other solitary organs. These data may be informative for physicians to identify patients presenting with higher probability of lung metastases and to be aware of the insult of lung metastases on survival outcome for PC patients.

## Figures and Tables

**Table 1:** Patient’s basic characteristics.

Characteristics	N (%)
Median age, year (range)	62 (25–88)
Sex	
male	393 (60.1)
female	261 (39.9)
Body mass index, Kg/m^2^, median (range)	22.4 (13.0–36.2)
Active or ever smoker	249 (38.1)
ECOG performance scale	
0 or 1	450 (68.8)
2	174 (26.6)
3	30 (4.6)
Charlson comorbidity index	
0	177 (27.1)
1	227 (34.7)
2	148 (22.6)
3	79 (12.1)
4	18 (2.8)
5	5 (0.8)
Body weight loss, %, median (range)	5 (0–33)
Primary tumor site ^+^	
head	224 (34.3)
body	121 (18.5)
tail	162 (24.8)
overlapping	147 (22.5)
Tumor size, cm, median (range) ^+^	4.5 (1.4–14.0)
Previous pancreatectomy	
yes	23 (3.5)
no	631 (96.5)
T-classification, 7th AJCC ^+^	
1	13 (2)
2	82 (12.5)
3	188 (28.7)
4	371 (56.7)
N-classification, 7th AJCC ^+^	
0	134 (20.5)
1	520 (79.5)
Number of metastatic organ ^+^	
1	391 (59.8)
2	191 (29.2)
3	53 (8.1)
≥4	19 (2.9)
Organ of metastases *^,^^+^	
liver	438 (67.0)
peritoneum	239 (36.5)
distant lymph nodes	150 (22.9)
lung	98 (15.0)
others	51 (6.1)
CEA, ng/dL	
≤5	290 (44.3)
>5	364 (55.7)
CA19-9, ng/dL	
≤37	130 (19.9)
>37	524 (80.1)
First-line chemotherapeutic regimen	
Gemcitabine monotherapy	208 (31.8)
Gemcitabine plus platinum or 5-fluorouracil/TS-1	398 (60.9)
5-fluorouracil +/- oxaliplatin +/- irinotecan	15 (2.3)
TS-1 monotherapy	32 (4.9)

ECOG, Eastern Cooperative Oncology Group; AJCC, American Joint Committee on Cancer; CEA, carcinoembryonic antigen; CA19-9, carbohydrate Antigen 19-9. * Some patients presented with multiple organ metastases; therefore, sum of percentage was over 100%. ^+^ The information acquired is based on image confirmed by a multidisciplinary tumor board.

**Table 2:** Univariate and multivariate analysis for presence with lung metastases.

Variable	Category	No of Lung Metastases/No of Total Patients (%)	Univariate Analysis		Multivariate Analysis	
			OR (95% CI)	*p* Value	Adjusted OR (95% CI)	*p* Value
Overall		98/654 (15.0)				
Sex	male	46/393 (11.7)	1		1	
	female	52/261 (19.9)	1.88 (1.22–2.89)	0.004	1.90 (1.23–2.95)	0.004
Age, year	≤65	39/309 (12.6)	1		1	
	>65	59/345 (17.1)	1.43 (0.93–2.21)	0.09	1.37 (0.88–2.14)	0.17
ECOG performance	0 or 1	65/450 (14.4)	1			
	2	27/174 (15.5)	1.09 (0.67–1.77)	0.74		
	3	6/30 (20)	1.48 (0.58–3.76)	0.41		
CCI	0 or 1	57/404 (14.1)	1			
	>1	41/250 (16.4)	1.19 (0.77–1.85)	0.43		
Active or ever smoking	no	63/405 (15.6)	1			
	yes	35/249 (14.1)	0.89 (0.57–1.39)	0.60		
CEA, ng/dL	≤5	46/290 (15.9)	1			
	>5	52/364 (14.3)	0.88 (0.58–1.36)	0.58		
CA19-9, ng/dL	≤37	22/130 (16.9)	1			
	>37	76/524 (14.5)	0.83 (0.49–1.41)	0.49		
Body weight loss	≤5%	40/286 (14.0)	1			
	>5%	47/287 (16.4)	1.20 (0.76–1.90)	0.43		
Primary tumor site	head	32/224 (14.3)	1			
	body	20/121 (16.5)	1.19 (0.65–2.18)	0.58		
	tail	24/162 (14.8)	1.04 (0.59–1.85)	0.88		
	overlapping	22/147 (15.0)	0.59–1.90	0.86		
Tumor grade	well to moderate	3/63 (4.8)	1		1	
	poorly or undifferentiated	95/591 (16.1)	3.83 (1.18–12.5)	0.026	4.08 (1.24–13.4)	0.020
T-classification	1 or 2	12/95 (12.6)	1			
	3	29/188 (15.4)	1.26 (0.61–2.60)	0.53		
	4	57/371 (15.4)	1.26 (0.64–2.45)	0.50		
N-classification	0	21/134 (15.7)	1			
	1	77/520 (14.8)	0.94 (0.55–1.58)	0.80		
T-size	<8 cm	86/609 (14.1)	1		1	
	≥8 cm	12/45 (26.7)	2.21 (1.10–4.45)	0.026	2.23 (1.09–4.54)	0.027

ECOG, Eastern Cooperative Oncology Group; CCI, Charlson comorbidity index; CEA, carcinoembryonic antigen; CA19-9, carbohydrate Antigen 19-9; OR, odds ratio.

**Table 3:** Univariate and multivariate analysis for overall survival.

Variable	Category	Univariate Analysis		Multivariate Analysis	
		HR (95% CI)	*p* Value	Adjusted HR (95% CI)	*p* Value
Sex	male	1		1	
	female	0.84 (0.71–0.99)	0.033	0.86 (0.72–1.01)	0.86
Age, year	≤65	1		1	
	>65	1.30 (1.10–1.52)	0.002	1.02 (0.86–1.22)	0.80
ECOG performance	0 or 1	1		1	
	2	2.50 (2.08–3.01)	<0.001	2.46 (2.02–2.99)	<0.001
	3	5.60 (3.75–8.36)	<0.001	5.79 (3.83–8.77)	<0.001
Body weight loss	≤5%	1			
	>5%	1.04 (0.87–1.23)	0.67		
Metastatic organ	isolated lung	1		1	
	isolated liver	1.96 (1.18–3.25)	0.010	1.92 (1.15–3.21)	0.013
	isolated peritoneum	1.68 (0.99–2.85)	0.055	1.54 (0.90–2.62)	0.11
	isolated lymph nodes	1.38 (0.75–2.55)	0.31	1.47 (0.79–2.74)	0.22
	multiple sites	2.66 (1.60–4.42)	<0.001	2.60 (1.56–4.35)	<0.001
CCI	0 to 1	1		1	
	>1	1.34 (1.40–1.58)	<0.001	1.21 (1.02–1.44)	0.027
Active or ever smoker	no	1		1	
	yes	1.16 (0.98–1.36)	0.080	1.22 (1.03–1.44)	0.021
CEA, ng/dL	≤5	1		1	
	>5	1.17 (0.99–1.38)	0.053	1.15 (0.98–1.36)	0.090
CA19-9, ng/dL	≤37	1			
	>37	1.03 (0.84–1.25)	0.81		

ECOG, Eastern Cooperative Oncology Group; CCI, Charlson comorbidity index; CEA, carcinoembryonic antigen; CA19-9, carbohydrate Antigen 19-9.
